# Intracellular Expression of CTB in *Vibrio cholerae* Strains in Laboratory Culture Conditions

**DOI:** 10.4014/jmb.2302.02014

**Published:** 2023-04-06

**Authors:** Hunseok Choi, Seonghyeon Son, Donghyun Lee, Jonghyun Bae, Eunyoung Seo, Dong Wook Kim, Eun Jin Kim

**Affiliations:** 1 Department of Pharmacy, College of Pharmacy, Hanyang University, Ansan 15588, Republic of Korea; 2 Institute of Pharmacological Research, Hanyang University, Ansan 15588, Republic of Korea

**Keywords:** Cholera, cholera toxin (CT), cholera toxin B subunit (CTB), *Vibrio cholerae*, vaccine

## Abstract

The introduction of the *toxT*-139F allele triggers the expression of TCP (toxin co-regulated pilus) and CT (cholera toxin) under simple laboratory culture conditions in most *Vibrio cholerae* strains. Such *V. cholerae* strains, especially strains that have been used in OCVs (oral cholera vaccines), can induce antibody responses against TCP in animal models. However, CT produced in these *V. cholerae* strains is secreted into the culture medium. In this study, *V. cholerae* strains that can express intracellular CTB under the control of the *toxT*-139F allele have been constructed for potential application in OCVs. First, we constructed a recombinant plasmid directly linking the *ctxAB* promoter to *ctxB* without *ctxA* and confirmed CTB expression from the plasmid in *V. cholerae* containing the *toxT*-139F allele. We constructed another recombinant plasmid to express NtrCTB, from which 14 internal amino acids—from the 7^th^ to the 20^th^ amino acid—of the leader peptide of CTB have been omitted, and we found that NtrCTB remained in the cells. Based on those results, we constructed *V. cholerae* strains in which chromosomal *ctxAB* is replaced by *ntrctxB* or *ntrctxB*-dimer. Both NtrCTB and NtrCTB-dimer remained in the bacterial cells, and 60% of the NtrCTB-dimer in the bacterial cells was maintained in a soluble form. To develop improved OCVs, these strains could be tested to see whether they induce immune responses against CTB in animal models.

## Introduction

Cholera is a severe diarrheal disease caused by a gram-negative bacterium, *Vibrio cholerae* [1]. The disease symptoms are caused by the cholera toxin (CT) that the bacteria produce and secrete in the human small intestine [2]. More than 200 serogroups of *V. cholerae* have been identified, and strains belonging to two serogroups—O1 and O139—harbor CT genes (*ctxAB*) and cause epidemic cholera [2]. O1 serogroup strains are further classified into classical and El Tor biotypes based on their microbiological characteristics [1]. Since the early 19^th^ century, seven cholera pandemics have been recognized. The first six of them were caused by classical biotype strains, and El Tor biotype strains are responsible for the current 7^th^ cholera pandemic, which began in 1961 [3, 4].

In addition to the action of CT, toxin co-regulated pilus (TCP) has been found to play important roles in the colonization of cholera bacteria in the human small intestine [2]. Thus, CT and TCP are considered to be the major virulence factors of *V. cholerae* [5]. The production of CT and TCP in *V. cholerae* is tightly regulated by the ToxR regulon [6, 7], through which the transcriptional activator ToxT stimulates the expression of CT and TCP. Recently, we have shown that a point mutation in *toxT*, *toxT*-139F, can trigger the expression of virulence genes in many *V. cholerae* strains with little or no environmental stimulation [8-11]. TCP expressed via the *toxT*-139F allele was shown to be functional as a receptor of CTXΦ infection and immunogenic when delivered to an animal model [10, 11].

CT expressed under laboratory culture conditions is secreted into culture medium by the type II secretion system [27]; thus CT production can be measured in culture supernatant [8, 12]. The secreted CT is the typical AB5 toxin composed of a single CTA (active subunit) and 5 CTB (binding subunit) polypeptides [1]. The *ctxB* ORF is 375-bp long, and the CTB consists of 124 a.a.; however, the first 21 amino acids are cleaved during secretion, so the mature CTB is composed of 103 amino acids [1, 2].

Recovered cholera patients have been shown to be protected against consecutive infection, and currently, oral cholera vaccines (OCVs) are recommended in endemic and epidemic areas [13, 14]. Inactivated OCVs (Shanchol and Euvichol) have been pre-qualified by the World Health Organization (WHO) for use in endemic areas. Another inactivated vaccine (Dukoral), which contains recombinant CTB (rCTB), has been pre-qualified by WHO and suggested for travelers [13, 15]. In the U.S., a live-attenuated OCV that can induce immunity against O-antigens and CTB has been licensed for travelers [13, 16].

Although current OCVs can provide satisfactory protective immunity against *V. cholerae* infection, improved vaccines are under development to ensure longer protective duration and better immunity in children in endemic areas [13, 15]. O-antigen-based subunit vaccines and several subunit vaccines based on CTB and other protein antigens are under development [18-21]. The expression of CTB in transgenic tomato plants for use in a cholera vaccine [22] and the immunogenicity of a rice-based CTB vaccine, MucoRice-CTB, have been reported [23]. Construction of a *V. cholerae* strain that expresses intracellular CTB by disrupting the bacterial type II secretion system and induction of anti-CTB responses in a mouse model using this engineered *V. cholerae* strain have also been reported [24]. We have shown that *V. cholerae* strains can be modulated to express functional and immunogenic TCP via the *toxT*-139F allele, and that approach could be introduced into *V. cholerae* strains that are already being used in OCVs [10].

In the work for this study, we developed *V. cholerae* strains that express intracellular CTB for potential use in OCVs. We designed recombinant plasmids that can be used to express CTB and variant CTBs under the control of the *ctxAB* promoter in *V. cholerae* strains, and we found that the variant CTBs (which is missing 14 amino acids from its 21 a.a.-long leader peptide) can remain in bacterial cells. To enable the intracellular expression of CTB variants from the CTX prophage on the chromosome, we constructed *V. cholerae* strains in which the *ctxAB* genes are replaced by variant *ctxB*— *ntrctxB*, or *ntrctxB*-dimer. The NtrCTB and NtrCTB-dimer expressed in those *V. cholerae* strains remained in the cells, and a significant portion of NtrCTB-dimer was maintained in the soluble cellular fraction. Therefore, these strains could be further examined for potential application in OCVs that could induce immune responses against CTB, in addition to O-antigens.

## Materials and Methods

### DNA Primers for Subcloning

DNA sequences and the location of the primers on the CTX phage genome used in this study are shown in Figs. S3 and S4.

### Plasmids and Bacterial Strains

The bacterial strains and recombinant plasmids used for the expression of CTB variants and the allele exchange experiments are shown in Table 1.

### Western Blot Analysis of Cholera Toxin

Anti-CT (C3062) and anti-CTB (ab34992) were purchased from Sigma-Aldrich (USA) and Abcam (UK), respectively. To prepare the culture fractions for the Western blot analyses, an overnight culture of *V. cholerae* strains was diluted to 10^9^ CFU/ml, and 200 μl of 4 × SDS sample buffer (250 mM Tris-Cl (pH 6.8), 8% SDS, 40%glycerol, 4% 2-mercaptoethanol, 0.02% bromophenol blue) were added to 600 μl of the diluted culture. To prepare the supernatant and cell fractions of the culture, 1 ml of the culture (diluted to 10^9^ CFU/ml) was centrifuged at 13,000 rpm for 10 min, and then the supernatant and cells were separated. 200 μl of 4 × SDS sample buffer was added to 600 μl of the supernatant. The cells were resuspended with 1 ml of 1 × SDS sample buffer (63 mM Tris-Cl (pH 6.8), 2% SDS, 10% glycerol, 0.1% 2-mercaptoethanol, 0.005% bromophenol blue). The culture, supernatant, and cell fractions samples were boiled for 5 min, and 10 μl of each fraction were analyzed on SDS-PAGE for the Western blot analysis.

### Construction of a *ctxB*-Expressing Isogenic Variant of the *V. cholerae* IB5230 Strain

An isogenic variant of IB5230 containing the *toxT*-139F allele, YJB020, has been shown to produce CT under simple laboratory culture conditions [8]. We constructed a *ctxB*-expressing isogenic variant of YJB020, EYS003, by deleting *ctxA* and linking the *ctxB* ORF to the promoter of *ctxAB* as follows. Using genomic DNA of IB5230, a 657-bp fragment from the 19^th^ nucleotide of *ctxB* to the 300^th^ nucleotide after the termination codon of *ctxB* was PCR-amplified using the primer pair ctxB-BamHI-XbaI F: GCC GGA TCC TCT AGA GGT GTT TTT TTT ACA GTT TTA C and ctxB-EcoRI R: GCC GAA TTC CAC AAT TGA CGT AAG TAC AG. This DNA fragment was digested with the restriction enzymes BamHI and EcoRI and inserted into the BamHI/EcoRI sites of a suicidal plasmid, pSW23-OriT [40], to construct pSW-ctxB. Using genomic DNA of IB5230, a 571-bp fragment encompassing a 442-bp fragment of *zot* (from the 759^th^ nucleotide to the termination codon of *zot*), a 105-bp fragment of the intergenic sequence between *zot* and *ctxA*, and the first 18 nucleotides of *ctxB* linked to a 6-bp restriction enzyme XbaI recognition sequence was PCR-amplified using the primer pair zot-SacI F: GGG GAG CTC GGG AAA TGA TGC AAC TAT CG and zot-XbaI R: CCC TCT AGA aaa ttt taa ttt aat cat ATA ATG CTC CCT TTG TTT AAC AG (the restriction enzyme XbaI recognition sequence is underlined, and the first 18 nucleotides of *cxB* are shown in lower-case letters). This fragment was digested with SacI/XbaI and inserted into the SacI/XbaI sites of pSW-ctxB to construct pSW-zot-ctxB. The recombinant suicidal plasmid pSW-zot-ctxB was conjugally transferred to EJK002 (an isogenic derivative of IB5230 in which *ctxAB* and the *toxT*-139Y allele are replaced by a kanamycin resistance cassette and *toxT*-139F, respectively). Then, EYS003, in which pSW-zot-ctxB was inserted in the chromosomal *zot* of EJK002, was selected among the conjugants.

### Construction of a Recombinant Plasmid that Expresses CTB Via the *ctxAB* Promoter

An 878-bp DNA fragment from EYS003, encompassing the last 92 nucleotides of *zot*, the 105 nucleotides of the intergenic sequence between *zot* and *ctxA*, and 681 nucleotides from the first nucleotide of *ctxB*, was PCR-amplified using the primer pair Pctx-HindIII F: CCC AAG CTT CCT TTG CAG CGC AAG CGC TG and ctxB-EcoRI R: GCC GAA TTC CAC AAT TGA CGT AAG TAC AG. This fragment was digested with HindIII/EcoRI and inserted into the HindIII/EcoRI sites of pUC18 to construct pUC18-ctxB. *E. coli* DH5α, *V. cholerae* EJK001, and EJK002 were transformed using pUC18-ctxB.

### Construction of a Recombinant Plasmid that Expresses Ntr*ctxB* Via the *ctxAB* Promoter

Using the genomic DNA of IB5230, a 565-bp DNA fragment encompassing a 547-bp fragment (from the 759^th^ nucleotide of *zot* to the end of the intergenic sequence between *zot* and *ctxA*) and the first 18 nucleotides of *ctxB* was PCR-amplified using the primer pair zot-sacI F: GGG GAG CTC GGG AAA TGA TGC AAC TAT CG and zot-XbaI R: CCC TCT AGA aaa ttt taa ttt aat cat ATA ATG CTC CCT TTG TTT AAC AG (the first 18 nucleotides of *ctxB* are shown in lower case). This fragment was digested with SacI and XbaI and inserted into pSW23-oriT to construct pSW23-zot. Using the genomic DNA of IB5230, a 621-bp fragment from the 61^st^ nucleotide of *ctxB* to the 300^th^ nucleotide after the termination codon of *ctxB* was PCR-amplified using the primer pair del-ctxB-XbaI F-1: GGG TCT AGA GGA ACA CCT CAA AAT ATT ACT and ctxB-EcoRI R: GCC GAA TTC CAC AAT TGA CGT AAG TAC AG . This DNA fragment was digested with XbaI and EcoRI and inserted into the XbaI/EcoRI site of pSW23-zot to construct the pSW23-zot-ntrctxB recombinant plasmid. Using the pSW23-zot-ntrctxB plasmid as the template, an 836-bp DNA fragment was PCR-amplified using the primer pair Pctx-HindIII F: CCC AAG CTT CCT TTG CAG CGC AAG CGC TG and ctxB-EcoRI R: GCC GAA TTC CAC AAT TGA CGT AAG TAC AG. This 836-bp fragment was digested with HindIII and EcoRI and inserted into HindIII/EcoRI sites on pUC18 to construct pUC18-ntrctxB.

### Construction of a Recombinant Plasmid that Expresses Ntr*ctxB*-Dimer Via the *ctxAB* Promoter

Using the genomic DNA of IB5230, a 312-bp DNA fragment from the 61^st^ nucleotide to the 372^nd^ nucleotide of *ctxB* was PCR-amplified using the primer pair del-ctxB-XbaI F-1: GGG TCT AGA GGA ACA CCT CAA AAT ATT ACT and ctxB-XbaI-BamHI R-1: CCC TCT AGA gga tcc ATT TGC CAT ACT AAT TGC (the BamHI restriction enzyme site shown in lower-case letters was designed for further insertion of the *ctxB* fragment). This DNA fragment was digested with XbaI and inserted into the XbaI site of pSW23-zot-ntrctxB to construct pSW23-zot-ntrctxB-dimer. Using the pSW23-zot-ntrctxB-dimer as the template, a 1,160-bp DNA fragment was PCR-amplified using the primer pair Pctx-HindIII F: CCC AAG CTT CCT TTG CAG CGC AAG CGC TG and ctxB-EcoRI R: GCC GAA TTC CAC AAT TGA CGT AAG TAC AG. This DNA fragment was digested with HindIII and EcoRI and inserted into the HindIII/EcoRI site of pUC18 to construct pUC18-ntrctxB-dimer.

### Construction of *V. cholerae* Strains that Express Ntr*ctxB* and Ntr*ctxB*-Dimer

A 939-bp DNA fragment from the 61^st^ nucleotide of *ctxB* to the last nucleotide of the intergenic sequence between *ctxB* and *rstR^ET^* on chromosome 1 of *V. cholerae* strain V212-1 was PCR-amplified using the primer pair del-ctxB-BamHI F-2: GGG GGA TCC GGA ACA CCT CAA AAT ATT ACT and ctxB-ig HindIII R Ch1: CCG AAG CTT GCG CAT CTT AAA TCA TGG TGC (Fig. S4). This DNA fragment was digested with BamHI and HindIII and inserted into the BamHI/HindIII sites of pUC18 to construct pUC18-ntrctxB-V. Using the genomic DNA of IB5230, a 544-bp DNA fragment from the 780^th^ nucleotide of *zot* to the last nucleotide of the intergenic sequence between *zot* and *ctxA* linked to the first 18 nucleotides of *ctxB* was PCR-amplified using the primer pair Zot-EcoRI F: CCG GAA TTC GCG TCA GAG CAA TCC GAG CCT and zot-BamHI R: CCC GGA TCC aaa ttt taa ttt aat cat ATA ATG CTC CCT TTG TTT AAC AG. This DNA fragment was digested with EcoRI and BamHI and inserted into the EcoRI/BamHI site of pUC18-ntrctxB-V to construct pUC18-zot-ntrctxB-V. A 1,489-bp DNA fragment was PCR-amplified from pUC18-zot-ntrctxB-V using the primer pair zot-XbaI F: CCG TCT AGA GCG TCA GAG CAA TCC GAG CCT and ctxB-ig-Uni-SacI R: CCG GAG CTC GCG CAT CTT AAA TCA TGG TGC. This DNA fragment was digested with XbaI and SacI and inserted into the XbaI/SacI site of a suicidal plasmid, pCVD442 [41], to construct pCVD-ntrctxB.

A 318-bp fragment of the second ntrctxB was PCR-amplified from the genomic DNA of IB5230 using the primer pair *ntrctxB* F2: GGG GGA TCC GGA ACA CCT CAA AAT ATT ACT and ctxB-BamHI-XhoI R: CCC GGA TCC ctc gag ATT TGC CAT ACT AAT TGC (the XhoI restriction enzyme site shown in lower-case letters was designed for the further insertion of a *ctxB* fragment). This fragment was digested with BamHI and inserted into the BamHI site of pUC18-zot-ntrctxB-V to construct pUC18-zot-ntrctxB-dimer-V. A 1,813-bp DNA fragment was PCR-amplified from the plasmid pUC18-zot-ntrctxB-dimer-V using the primer pair zot-XbaIF: CCG TCT AGA GCG TCA GAG CAA TCC GAG CCT and ctxB-ig-Uni-SacI R: CCG GAG CTC GCG CAT CTT AAA TCA TGG TGC. This DNA fragment was digested with XbaI and SacI and inserted into the XbaI/SacI site of the suicidal plasmid pCVD442 to construct pCVD-ntrctxB-dimer. pCVD-ntrctxB and pCVD-ntrctxB-dimer were conjugally transferred to YJB020 to construct the HSC001 and HSC002 strains, respectively

### Preparation of a Soluble Cytoplasmic Protein Fraction to Quantify NtrCTB-Dimer

*V. cholerae* strains were cultured for 16 h (O395 as a positive control and N16961 as a negative control were cultured at 30°C in 12 ml of LB medium containing 50 mM Tris at pH 6.5; HSC002, which expresses NtrCTB-dimer, was cultured in AKI broth at 37°C), and then the bacterial cells were counted using a spectrophotometer. Bacterial cells from 10 ml of culture were harvested by centrifugation, and the supernatant was filtered with a 0.2 μm filter and used to measure the secreted CT. The cells were resuspended in 10 ml of 1 × PBS and disrupted by the freeze–thaw method and subsequent sonication. The soluble cytoplasmic fractions were obtained from the disrupted cells by centrifugation (13,000 rpm for 10 min) and serially diluted (1/2, 1/4, 1/8, 1/16. 1/32. 1/64, and 1/ 100) for ELISA and Western blot analyses. CTB-dimer in insoluble fractions was analyzed by Western blotting. Western blot images were analyzed using an Odyssey CLx imaging system (LI-COR Biosciences, Lincoln, NE, USA). The Western blot band intensities representing TcpA were quantified using the ImageJ gel analysis program and LI-COR Odyssey software.

### Sandwich ELISA to Determine the Concentration of Intracellular NtrCTB-Dimer

The wells of transparent 96-well microtiter plates were coated with 100 μl of mouse anti-CTB (Abcam 35988, diluted 1: 1,000 in PBS) for 16 h at 4°C. The wells were then washed with 1 × PBST three times and blocked with blocking buffer (1% BSA in 1 × PBS) for 1.5 h. Soluble cytoplasmic fractions prepared as described above (100 μl) were added to each well and incubated for 2 h. The samples were removed, and the wells were washed three times; then, 100 μl of the primary antibody (rabbit anti-CTB, Abcam ab34992) 1/2,000 diluted in 1 × PBS were added. After 1 h of incubation, the primary antibody was removed, and the wells were washed three times. Then the secondary antibody (goat anti-rabbit IgG (HRP), GeneTex GTX213110-01) 1/5,000 diluted in 1 × PBS was added. The secondary antibody was removed, and the wells were washed three times, and then TMB solution was added. After adding the stop solution, we measured the samples using a plate reader (TECAN, Infinite 200 PRO) at O.D._450_.

## Results

### CT Is Produced and Secreted in *V. cholerae* Strains that Harbor the *toxT*-139F Allele

IB5230, a *V. cholerae* strain from a 2010 outbreak in Haiti, has been reported to be hypervirulent because it produces an unusually large amount of CT and hemolysin [25, 26]. Moreover, this strain was shown to produce CT at 37°C in PBS-buffered LB medium or AKI broth, whereas most El Tor biotype strains do not produce CT under those single-phase culture conditions [8]. When the *toxT*-139F allele was introduced to this strain (YJB020), CT was produced not only at 37°C but also at 30°C without the need for specific stimuli [8]. Therefore, in this study, we examined intracellular CT production in isogenic strains derived from IB5230 or YJB020.

**Fig. 1:**
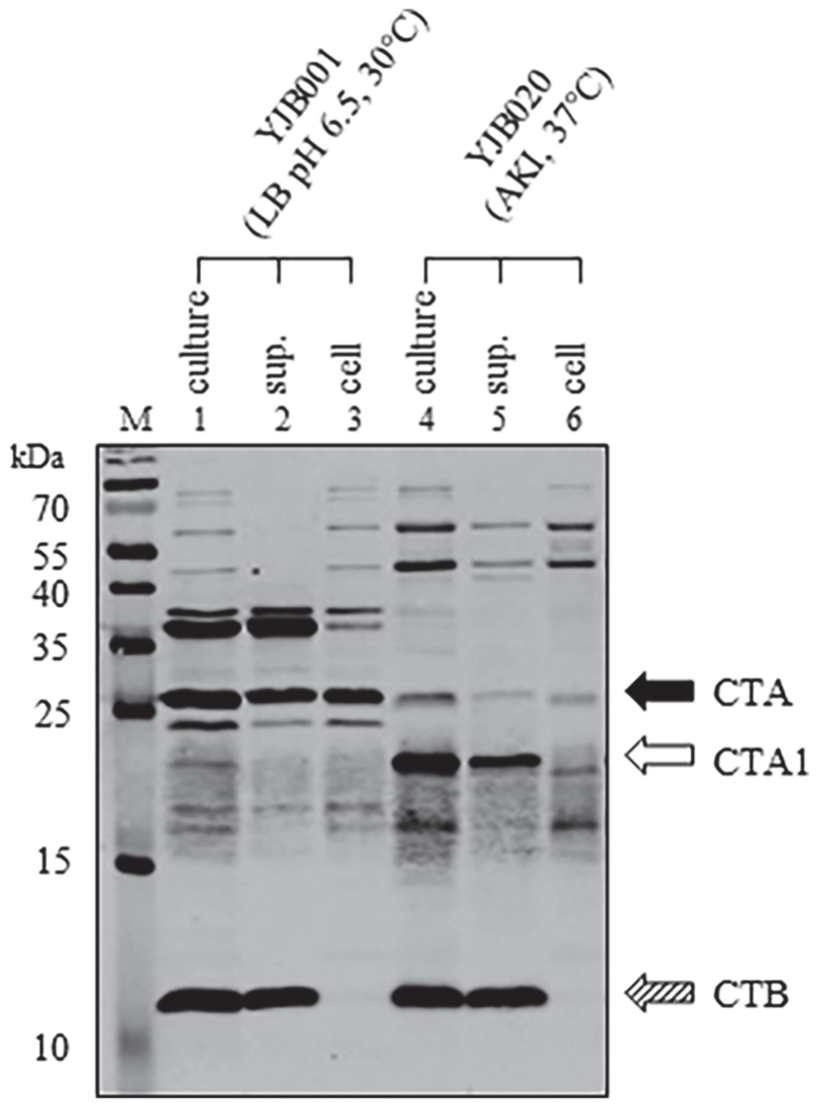
Secretion of cholera toxin in *V. cholerae* strains. The whole culture (culture), culture supernatant (sup.), and cell fractions (cell) were prepared as described in the Methods and analyzed by Western blotting using anti-CT. The CT expression of two *V. cholerae* strains, YJB001 and YJB020, which are *toxT*-139F derivatives of the classical biotype strain O395 and the Wave 3 El Tor biotype strain IB5230, respectively, was analyzed. CTA, CTA1, and CTB are indicated by black, white, and shaded arrows, respectively.

The secretion of CT from YJB001, a *toxT*-139F derivative of the classical biotype strain O395, and YJB020 was examined (Fig. 1). Bacteria were cultured in CT-producing conditions (YJB001 was cultured at 30°C in LB medium pH-adjusted to 6.5, and YJB020 was cultured at 37°C in AKI broth), and the whole culture, culture supernatant, and harvested cells were analyzed by Western blotting with anti-CT to examine the secretion of CT. In the classical biotype strain, YJB001, most CTB was secreted into the culture medium, though significant fractions of CTA remained in the bacterial cells (Fig. 1 lanes 1–3). The CT secretion pattern in the YJB020 strain was similar to that of YJB001 (Fig. 1 lanes 4–6). The CTA in YJB020 seemed to be cleaved into CTA1, whereas it remained intact in YJB001. Cleavage of CTA was previously reported in other *V. cholerae* strains [8]; however, the exact nature of that cleavage remains to be investigated.

### Construction of *ctxAB*-Deleted Variant of IB5230 and YJB020

EJK001(an isogenic derivative of IB5230 in which *ctxAB* has been replaced by a kanamycin-resistance cassette) and EJK002 (an isogenic variant of EJK001 in which the *toxT*-139Y allele has been replaced by the *toxT*-139F allele) were used in this study to ensure that no endogenous CTB was expressed [10]. Impairment of CT production in the strains was confirmed by Western blotting with anti-CT (Fig. S1). IB5230 and YJB020 (the *toxT*-139Y of IB5230 has been replaced by the *toxT*-139F allele) produced CT when cultured in AKI broth or LB medium at 37°C, and the CT production of IB5230 in LB medium was reduced to approximately 30% of that produced in AKI broth, as previously reported (Fig. S1. lanes 1–4) [8]. CT was not produced in EJK001 or EJK002 in any culture conditions (Fig. S1 lanes 5–8). EJK001 and EJK002 were therefore transformed with recombinant plasmids that express the *ctxB* variants described below.

### Expression of CTB from a Recombinant Plasmid Using the *ctxAB* Promoter (*P_ctxAB_*) in *V. cholerae* Strains

**Fig. 2:**
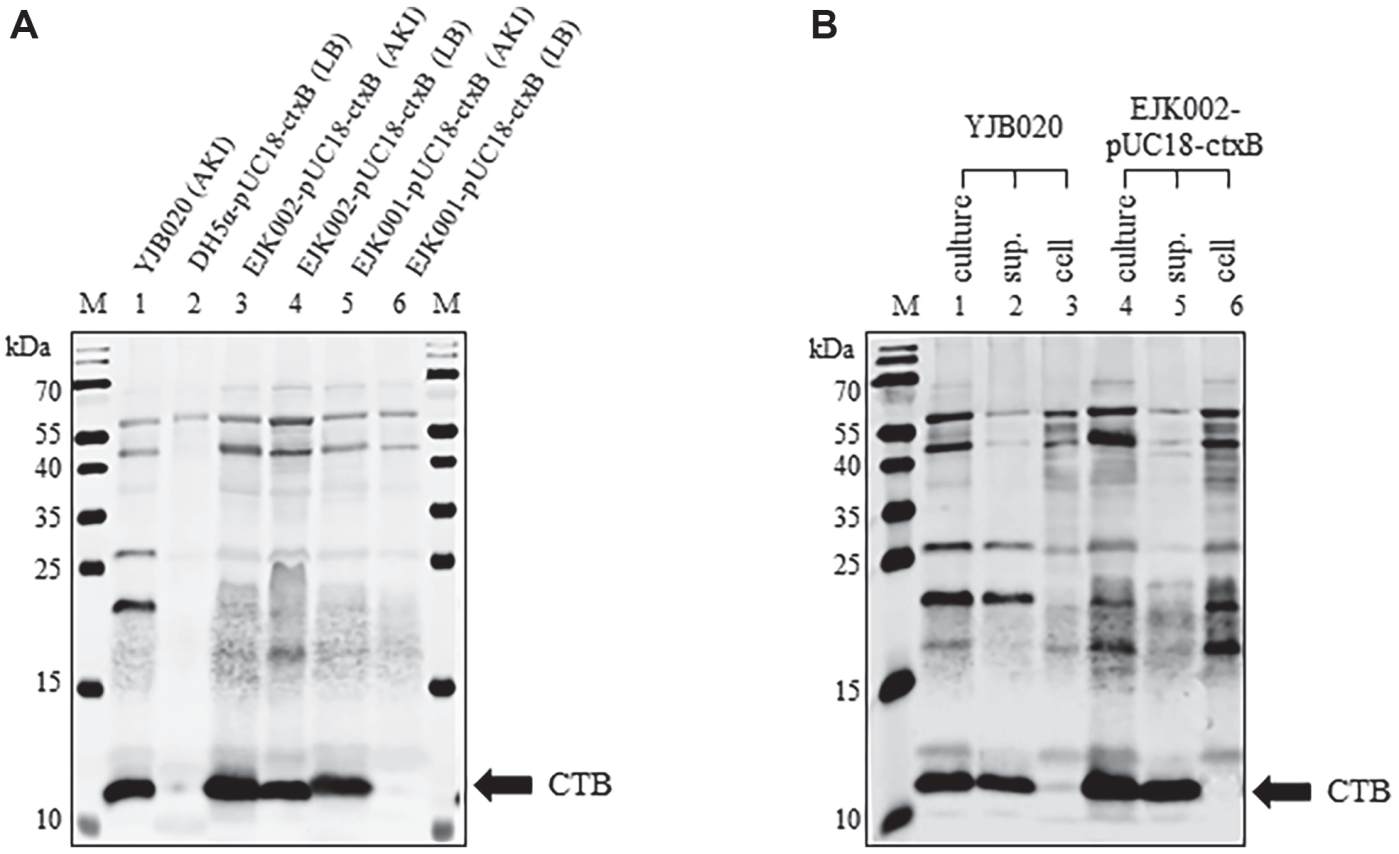
Expression of CTB from a recombinant plasmid that contains the *ctxB* ORF linked to the *ctxAB* promoter. (**A**) Expression of CTB in YJB020 (lane 1), DH5α-pUC18-ctxB (lane 2), EJK002-pUC18-ctxB cultured in AKI broth (lane 3), EJK002-pUC18-ctxB cultured in LB medium (lane 4), EJK001-pUC18-ctxB cultured in AKI broth (lane 5), and EJK001-pUC18-ctxB cultured in LB medium (lane 6). Bacteria were cultured at 37°C. (**B**) Secretion of CTB from *V. cholerae* strains that express CTB from pUC18-ctxB (EJK002-pUC18-ctxB). Culture (lanes 1 and 4), culture supernatant (lanes 2 and 5), and cells (lanes 3 and 6) of YJB020 and EJK002-pUC18-ctxB were analyzed. Bacteria were cultured in AKI broth at 37°C.

*E. coli* strain DH5α and *V. cholerae* EJK001 and EJK002 were transformed with pUC18-ctxB, which was constructed to express CTB by means of the *ctxAB* promoter (*P_ctxAB_*) in *V. cholerae* (Fig. S2A). CTB was not produced in DH5α-pUC18-ctxB because the ORF of *ctxB* was inserted in the opposite direction of the lac promoter of the pUC18 vector, and the *ctxAB* promoter cannot be used in *E. coli* (Fig. 2A lane 2). CTB was expressed by pUC18-ctxB in EJK002 cultured in AKI broth or LB medium at 37°C (Fig. 2A lanes 3 and 4), indicating that the *ctxB* ORF linked to the *ctxAB* promoter on the recombinant plasmid was transcribed. CTB was also expressed by pUC18-ctxB in EJK001 cultured in AKI medium, while the expression level was low when cultured in LB medium (Fig. 2A lanes 5 and 6).

CTB produced in EJK002-pUC18-ctxB was examined to see whether it was secreted into the medium. Just as the endogenous CTB produced in YJB020 was secreted into the culture medium (Fig. 2B lanes 1–3), CTB produced in EJK002-pUC18-ctxB was also secreted into the medium (Fig. 2B lanes 4–6). These results indicate that the *ctxB* linked to the *ctxAB* promoter on the recombinant plasmid is transcribed similarly to chromosomal *ctxAB* and that the CTB produced from the recombinant plasmid is secreted into the medium.

### Expression of NtrCTB and NtrCTB-Dimer from Recombinant Plasmids Using the *P_ctxAB_* in *V. cholerae*

**Fig. 3:**
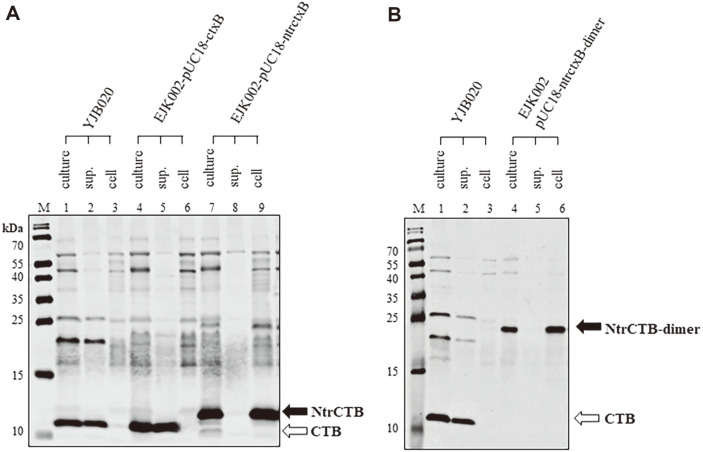
Expression of CTB variants in *V. cholerae* strains that harbor pUC18-ctxB, pUC18-ntrctxB, or pUC18- ntrctxB-dimer. (**A**) Expression of CTB and NtrCTB in *V. cholerae* strains harboring pUC18-ctxB and pUC18-ntrctxB, respectively. Culture (lanes 1, 4, and 7), culture supernatant (lanes 2, 5, and 8), and cells (lanes 3, 6, and 9) of YJB020 (lanes 1–3), EJK002-pUC18-ctxB (lanes 4–6), and EJK002-pUC18-ntrctxB (lanes 7, 8, and 9) that were cultured in AKI broth at 37°C were analyzed by Western blotting using anti-CT. CTB and NtrCTB are indicated by white and black arrows, respectively. (**B**) Expression of CTB and NtrCTB-dimer in *V. cholerae* strains that harbor pUC18-ctxB-dimer. Culture (lanes 1 and 4), culture supernatant (lanes 2 and 5), and cells (lanes 3 and 6) of YJB020 (lanes 1–3) and EJK002-pUC18-ntrctxB-dimer (lanes 4–6) that were cultured in AKI broth at 37°C were analyzed. CTB and NtrCTB-dimer are indicated by white and black arrows, respectively.

Because the *ctxB* linked to the *ctxAB* promoter in a recombinant plasmid could be expressed in *V. cholerae* strains containing the *toxT*-139F allele, we constructed two more recombinant plasmids, which express NtrCTB and NtrCTB-dimer, to examine whether the CTB would remain in the cell when 14 amino acids from the N-terminal leader peptide were omitted (construction methods are described in the Materials and Methods section). NtrCTB and NtrCTB-dimer were expressed in *V. cholerae* EJK002 strains harboring pUC18-ntrctxB and pUC18-ntrctxB-dimer, respectively (Figs. S2B, S2C, and 3). The molecular weight of NtrCTB (12.3 kDa) produced in EJK002-pUC18-ntrctxB was higher than that of authentic CTB (11.3 kDa, 103 a.a.) produced in YJB020 and EJK002-pUC18-ctxB because the first 21 amino acids of CTB have been cleaved. In contrast, NtrCTB is composed of 112 a.a.—the first 6 amino acids of *ctxB*, 2 amino acids encoded by a restriction enzyme site that was inserted during the cloning process, and 104 amino acids of CTB (from the 21^st^ to the 124^th^ amino acid). In NtrCTB-dimer (218 a.a.), two amino acids encoded by a restriction enzyme site inserted during the subcloning process and 104 amino acids of CTB have been added to NtrCTB; therefore, the molecular weight of NtrCTB-dimer was approximately 24 kDa. Whereas the authentic CTB produced in YJB020 and the CTB expressed from pUC18-ctxB were secreted into the medium (Fig. 3A lanes 1–6), the NtrCTB and NtrCTB-dimer remained in the cells (Figs. 3A lanes 7–8 and 3B lanes 4–6). These results indicate that NtrCTB and NtrCTB-dimer can be expressed from the *ctxAB* promoter and remain in the *V. cholerae* cells.

### Expression of CTB Variants Integrated into the Chromosome of *V. cholerae*

**Fig. 4:**
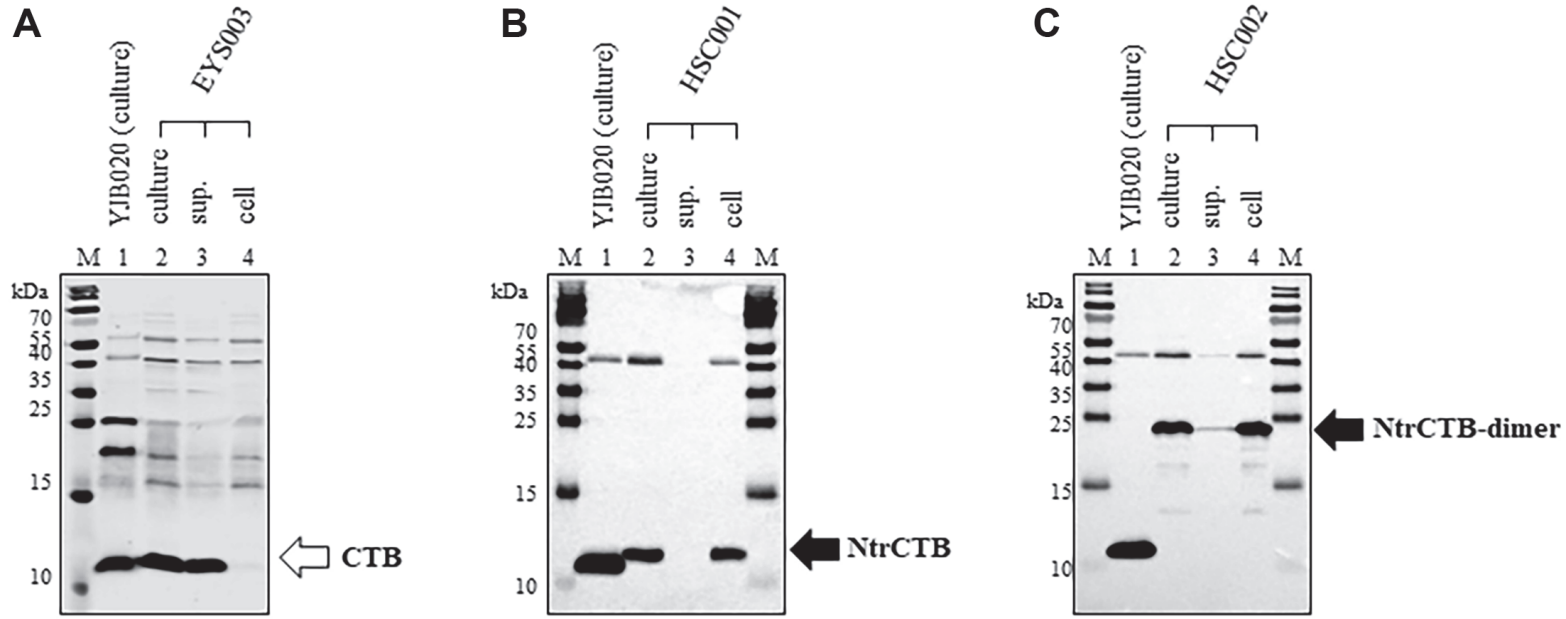
Expression of CTB variants in *V. cholerae* strains in which *ctxB*, *ntrctxB*, and ntrctxB-dimer were integrated into the chromosome. The expression of variant CTB was analyzed by Western blotting with anti-CT (**A**) and anti-CTB (**B** and **C**). (**A**) EYS003 expressing CTB, (**B**) HSC001 expressing NtrCTB, and (**C**) HSC002 expressing NtrCTB-dimer. M: molecular weight marker, lane 1: culture of YJB020, lane 2: culture, lane 3: culture supernatant, and lane 4: cells of each strain.

**Fig. 5:**
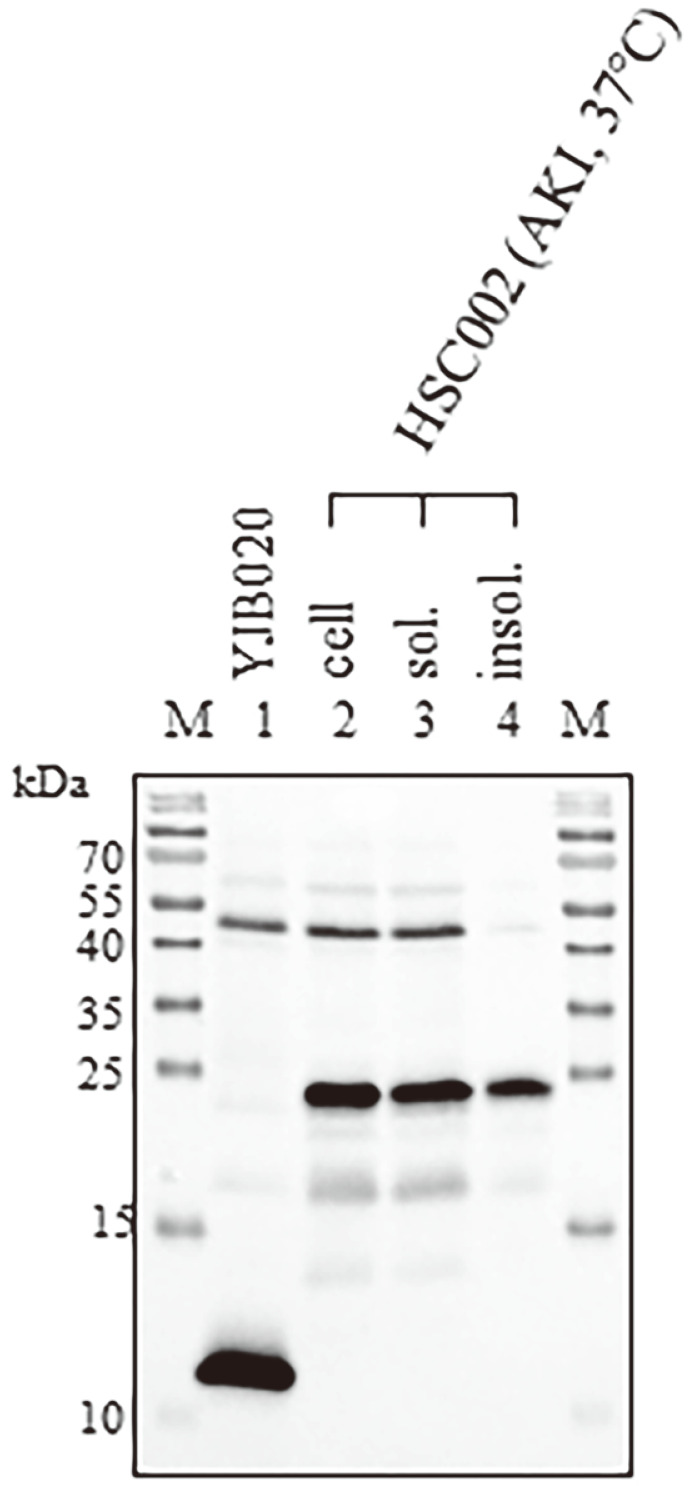
CTB dimer expressed in *V. cholerae* strain remains in soluble fractions. YJB020 and HSC002 were cultured in AKI broth at 37°C. Then, the whole culture of YJB020 (lane 1), harvested cells of HSC002 (lane 2), soluble fraction (lane 3), and insoluble fraction (lane 4) of HSC002 were analyzed by Western blotting using anti-CTB.

Because the *ctxB*, *ntrctxB*, and *ntrctxB*-dimer that were linked to the *ctxAB* promoter on the recombinant plasmids could be expressed similarly as authentic chromosomal *ctxB*, we anticipated that the *ctxAB* of YJB020 could be replaced by *ctxB*, *ntrctxB*, or *ntrctxB*-dimer and then express the *ctxB* variants from the chromosome. To test that expectation, we constructed *V. cholerae* strains EYS003, HSC001, and HSC002, which express CTB, NtrCTB, and NtrCTB-dimer, respectively (construction methods are described in the Materials and Methods section). As expected, CTB was produced and secreted from EYS003 cultured at 37°C in AKI broth (Fig. 4A), and the NtrCTB and NtrCTB-dimer produced by HSC001 and HSC002 remained in the cells (Figs. 4B and 4C). Moreover, more than 60% of the CTB-dimer produced in HSC002 remained in the soluble fraction of the cells (Fig. 5). We also measured the CTB-dimer in the soluble fraction of HSC002 using sandwich ELISA (described in the Materials and Methods) and found that 1.2 μg of soluble intracellular CTB-dimer could be produced from 10^11^ cells of HSC002. On the other hand, the amount of CT secreted by IB5230 into the culture supernatant was approximately 450 μg per 10^11^ cells.

## Discussion

WHO recommends the use of OCVs to control endemic and epidemic cholera [28]. Two types of killed OCVs that commonly contain three O1 serogroup strains and an O139 serogroup strain (Shanchol and Euvichol) or recombinant CTB (Dukoral) have been pre-qualified by WHO [13]. Vaccines that contain only killed cells are expected to induce immune responses against O-antigens of lipopolysaccharide (LPS) from the bacteria, and the price for the public sector has been deemed reasonable (1–1.85 USD/dose) for developing countries [13].

The induction of immune responses against O-antigens and CTB have also been reported for Dukoral, which contains rCTB, but the price of Dukoral is higher (4.7–9.4 USD/dose) than that of the killed-cells only OCVs due to the production cost of the rCTB (1 mg of rCTB is included in a single dose of Dukoral, which also contains 1.25 × 10^11^ bacterial cells) [13]. Immune responses against CTB have been shown to be helpful against the heat-labile toxin (LT) of enterotoxigenic *E. coli* (ETEC) due to immunological cross-reactivity between CTB and LT [15].

The expression of CT and TCP is tightly regulated by the ToxR regulon during *V. cholerae* infection [31]. To understand the toxigenicity of *V. cholerae*, various culture conditions have been developed to induce CT and TCP in *V. cholerae* under laboratory culture conditions [32-34]. Agglutinating culture conditions in which the bacteria are cultured in LB medium (pH adjusted at 6.5 by 50 mM Tris-Cl) at 30°C have been widely applied to induce CT and TCP production in *V. cholerae* O1 serogroup classical biotype strains [35], whereas more complicated culture conditions, *i.e.*, AKI culture conditions or shallow culture, are required to induce virulence genes in El Tor biotype strains [36, 37]. However, the *V. cholerae* strains used in OCVs are not cultured in any of those virulence gene–induction conditions during the manufacturing process; thus, only immune responses against O-antigens are anticipated after exposure to killed-cell-based OCVs. Moreover, evaluation of the efficacy of cholera vaccines has been determined using only a surrogate model—the vibriocidal assay—because no appropriate animal models have been developed for cholera, and the relevance of the vibriocidal assay to vaccine efficacy has been reported [38]. However, antibody responses against other bacterial components still need to be validated more thoroughly.

Although the O-antigens on LPS and CTB are important antigens for providing protective immunity against consecutive *V. cholerae* infection [15], other cellular components, such as TCP, sialidase, and flagellins, have also been shown to induce antibody responses during *V. cholerae* infection [17].

We recently reported that the *V. cholerae* strains used in OCVs could be improved by using endogenously expressed TCP via the *toxT*-139F allele to induce antibody responses against TCP when delivered by the intraperitoneal route [10]. In the work for this study, we constructed *V. cholerae* El Tor biotype strains that express intracellular CTB and CTB-dimer under the control of the *toxT*-139F allele. CT—at least CTB—produced by *V. cholerae* strains is secreted [39], and the N-terminal 21-amino-acid leader peptides of CTB are removed during the secretion process [1]. We therefore connected a truncated *ctxB* (omitting 16 amino acids from the leader peptides) to the *ctxAB* promoter and confirmed that the resulting NtrCTB remained in the cells. We also confirmed that NtrCTB-dimer could be expressed intracellularly in soluble form.

One milligram of rCTB together with 1.25 × 10^11^ inactivated bacterial cells are required for a single dose of Dukoral [15]. The amount of intracellular CTB-dimer was 1/100–1/500 of the amount of secreted CTB reported for *V. cholerae* strains. Nonetheless, the bacterial strains that express intracellular CTB need to be examined to determine whether they can induce antibody responses against CTB in animal models because several intracellular enzymes from *V. cholerae* strains, such as phosphoenolpyruvate-protein phosphotransferase and diaminobutyrate-2-oxoglutarate aminotransferase, have been reported to induce such responses [17]. The *V. cholerae* strains presented here might also be further developed for the intracellular expression of a CTB pentamer mimicking intact, secreted CTB. The intracellular expression of CTB in classical biotype strains, as well as *V. cholerae* strains included in OCVs, could also be developed further.

## Supplemental Materials

Supplementary data for this paper are available on-line only at http://jmb.or.kr.

## Figures and Tables

**Table 1:** Plasmids and bacterial strains.

Plasmids	Description	Genome sequence information and reference
CTB Expression		
pUC18-ctxB	Expression of CTB under the control of the *ctxAB* promoter in *V. cholerae*	
pUC18-NtrctxB	Expression of NtrCTB under the control of the *ctxAB* promoter in *V. cholerae*	
pUC18-NtrctxB-dimer	Expression of NtrCTB-dimer under the control of the *ctxAB* promoter in *V. cholerae*	
Allele exchange		
pSW-zot-ctxB	*ctxB* is directly linked to *ctxAB* promoter for expression of CTB	
pCVD-ntrctxB	allele exchange vector for replacing chromosomal *ctxAB* with *ntrctxB*	
pCVD-ntrctxB-dimer	allele exchange vector for replacing chromosomal *ctxAB* with *ntrctxB*-dimer	
Bacterial strains		
O395	Classical biotype strain, *toxT*-139Y	
YJB001 (O395-*toxT*-139F)	*toxT*-139F	Baek *et al*., [8]
V212-1	Wave 2 El Tor variant	ERS013132 [43]
MG116025	Wave 2 El Tor variant , intrinsic *toxT*-139F	ERS013135 [43]
IB5230	Wave 3 El Tor variant (2010 Haitian outbreak), *toxT*-139Y	AELH00000000.1[42]
YJB020	*toxT*-139F variant of IB5230	Baek *et al*. [8]
EJK001	*ctxAB* of IB5230 was replaced by a kanamycin resistance cassette	Kim *et al*., [10]
EJK002	*toxT*-139F variant of EJK002	Kim *et al*., [10]
EYS003	*ctxA*-deleted YJB020 for chromosomal expression of *ctxB*	This study
HSC001	Variant of YJB020 that expresses NtrctxB	This study
HSC002	Variant of YJB020 that expresses NtrctxB-dimer	This study
