# Supplementary material for: Intracellular Expression of CTB in Vibrio cholerae Strains in Laboratory Culture Conditions
Source: J Microbiol Biotechnol. 2023 Apr 6;33(6):736–44. doi: 10.4014/jmb.2302.02014 (PMC10331945; doi:10.4014/jmb.2302.02014)
Supplement: Supplementary file 1 [file jmb-33-6-736-supple.pdf]

Fig. S1.

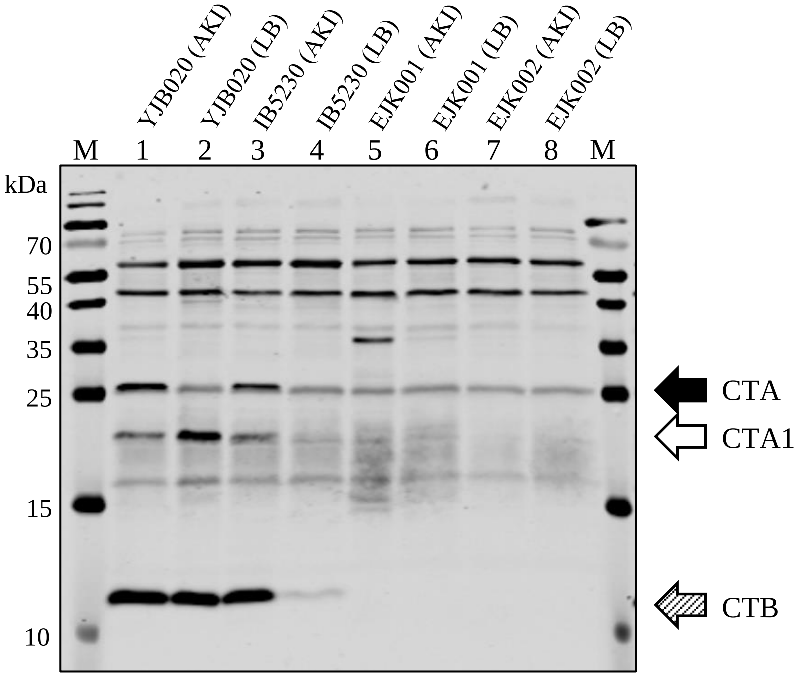
YJB020 (AKI)
YJB020 (LB)
IB5230 (AKI)
IB5230 (LB)
EJK001 (AKI)
EJK001 (LB)
EJK002 (AKI)
EJK002 (LB)
M 1 2 3 4 5 6 7 8 M
kDa
70
55
40
35
25
15
10
CTA
CTA1
CTB

Fig. S2.

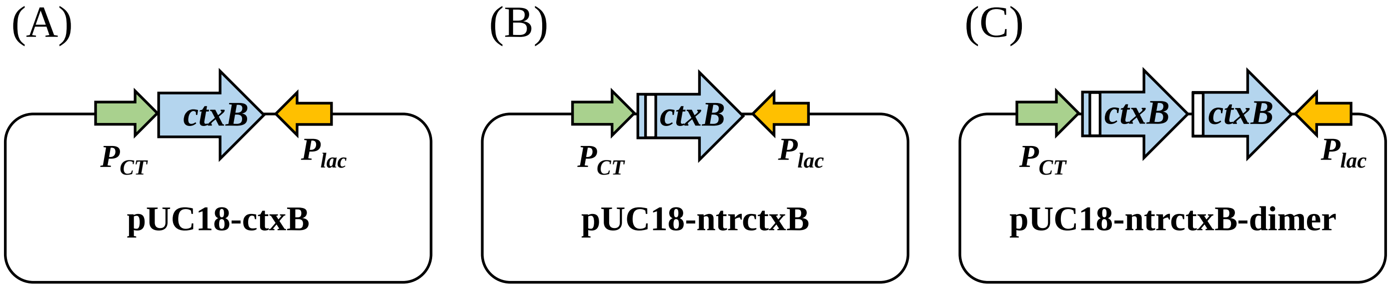
(A)
ctxB
$P_{CT}$
$P_{lac}$
pUC18-ctxB
(B)
ctxB
$P_{CT}$
$P_{lac}$
pUC18-ntrctxB
(C)
ctxB
ctxB
$P_{CT}$
$P_{lac}$
pUC18-ntrctxB-dimer

# Fig. S3.

GCAAAGCACG CGACACGATG GCCGGAACGG CGCTGTGGAA AGACAGAAAG ATCCTTTTCT
TGTTCGGCAT GGTTTTTTTG ATGTTCTCTT ATTCGTTTTA CGGCTTACAC GACAATCCAA
zot-SacI F TTTTTACAGG GGGAAATGAT GCAACTATCG AGTCAGAGCA ATCCGAGCCT CAGTCAAAGG Zot-EcoRI F Zot-XbaI F
CTACTGTTGG GAATGCTGTC GGGAGCAAGG CGGTTGCTCC TGCGTCTTTT GGTTTTTGTA
TTGGTCGGCT TTGTGTCCAA GATGGTTTTG TCACTGTTGG TGATGAGCGT TATCGCCTCG
TAGACAATTT GGACATTCCT TATCGTGGTC TATGGGCGAC AGGTCATCAC ATTTACAAGG
ATACGCTTAC AGTGTTTTTT GAAACCGAGA GTGGCAGCGT CCCAACAGAG CTGTTTGCAT
CGAGCTACCG CTACAAGGTG CTACCGTTAC CGGATTTCAA TCACTTTGTG GTGTTCGATA
Pctx-HindIII F CCTTTGCAGC GCAAGCGCTG TGGGTAGAAG TGAAACGGGG TTTACCGATA AAAACAGAAA
ATGATAAAAA AGGACTAAAT AGTATATTTT GATTTTTGAT TTTTGATTTT TGATTTTTGA
TTTCAAATAA TACAAATTTA TTTACTTATT TAATTGTTTT GATCAATTAT TTTTCTGTTA
zot-XbaI R zot-BamHI R AACAAAGGGA GCATTATATG GTAAAGATAA TATTTGTGTT TTTTATTTTC TTATCATCAT
TTTCATATGC AAATGATGAT AAGTTATATC GGGCAGATTC TAGACCTCCT GATGAAATAA
AGCAGTCAGG TGGTCTTATG CCAAGAGGAC AGAGTGAGTA CTTTGACCGA GGTACTCAAA
TGAATATCAA CCTTTATGAT CATGCAAGAG GAACTCAGAC GGGATTTGTT AGGCACGATG
ATGGATATGT TTCCACCTCA ATTAGTTTGA GAAGTGCCCA CTTAGTGGGT CAAACTATAT
TGTCTGGTCA TTCTACTTAT TATATATATG TTATAGCCAC TGCACCCAAC ATGTTTAACG
TTAATGATGT ATTAGGGGCA TACAGTCCTC ATCCAGATGA ACAAGAAGTT TCTGCTTTAG
GTGGGATTCC ATACTCCCAA ATATATGGAT GGTATCGAGT TCATTTTGGG GTGCTTGATG
AACAATTACA TCGTAATAGG GGCTACAGAG ATAGATATTA CAGTAACTTA GATATTGCTC
CAGCAGCAGA TGGTTATGGA TTGGCAGGTT TCCCTCCGGA GCATAGAGCT TGGAGGGAAG
AGCCGTGGAT TCATCATGCA CCGCCGGGTT GTGGGAATGC TCCAAGATCA TCGATGAGTA
ATACTTGCGA TGAAAAAACC CAAAGTCTAG GTGTAAAATT CCTTGACGAA TACCAATCTA
AAGTTAAAAG ACAAATATTT TCAGGCTATC AATCTGATAT TGATACACAT AATAGAATTA
AGGATGAATT **atgattaaat taaaatttGG TGTTTTTTTT ACAGTTTTAC TATCTTCAGC** ctxB-BamHI-XbaI F
del-ctxB-XbaI F-1 del-ctxB-BamHI F-2 **ATATGCAAAT GGAACACCTC AAAATATTAC TGATTTGTGT GCAGAATACC ACAACACACA**
**AATACATACG CTAAATGATA AGATATTTTC GTATACAGAA TCTCTAGCTG GAAAAAGAGA**
**GATGGCTATC ATTACTTTTA AGAATGGTGC AACTTTTCAA GTAGAAGTAC CAGGTAGTCA**
**ACATATAGAT TCACAAAAAA AAGCGATTGA AAGGATGAAG GATACCCTGA GGATTGCATA**
**TCTTACTGAA GCTAAAGTCG AAAAGTTATG TGTATGGAAT AATAAAACGC CTCATGCGAT**
ctxB-XbaI-BamHI R-1 ctxB-BamHI-XhoI R **TGCCGCAATT AGTATGGCAA ATTAA**GATAT AAAAAAGCCC ACCTCAGTGG GCTTTTTTGT
GGTTCGATGA TGAGAAGCAA CCGTTTTGCC CAAACATGTA TTACTGCAAG TATGATGTTT
TTATTCCACA TCCTTAGTGC GTATTATGTA TGTTATGTTA AATTAAGGCA TAAAAAGAGG
TCGCAAACCC CAATCTGCTC CCTATTCTTA ATCCAGCATT AAACATAACT GTATTTACCA
TAAAATACCT AATAATCATA TTTATCATTT GACAATGTTA AGTATGATTA TTAGTGACAT
ctxB-EcoRI R TGTCACTGTA CTTACGTCAA TTGTGTGCAC CAAAGTGTTT CTGTAATCCC TTGATTTGAA
AACCTGTTAC CAACCTTTCA AATGTGTTTA GGTTTGCTTC GCTAAAAAAG AACTCCACAC
TAAGGCGGAG TTCTTGGATA TTTTGGCAGC TTGTTTTTGC TGCTTAAATT TGGGCTACGC

# Fig. S4.

CAGATGGTTA TGGATTGGCA GGTTTCCCTC CGGAGCATAG AGCTTGGAGG GAAGAGCCGT

GGATTCATCA TGCACCGCCG GGTTGTGGGA ATGCTCCAAG ATCATCGATG AGTAATACTT

GCGATGAAAA AACCCAAAGT CTAGGTGTAA AATTCCTTGA CGAATACCAA TCTAAAGTTA

AAAGACAAAT ATTTTCAGGC TATCAATCTG ATATTGATAC ACATAATAGA ATTAAGGATG

AATT**ATGATT AAATTAAAAT TTGGTGTTTT TTTTACAGTT TTACTATCTT CAGCATATGC**

del-ctxB-BamHI F-2 **ACATGGAACA CCTCAAAATA TTACTGATTT GTGTGCAGAA TACCACAACA CACAAATATA**

**TACGCTAAAT GATAAGATAT TTTCGTATAC AGAATCTCTA GCTGGAAAAA GAGAGATGGC**

**TATCATTACT TTTAAGAATG GTGCAATTTT TCAAGTAGAA GTACCAGGTA GTCAACATAT**

**AGATTCACAA AAAAAAGCGA TTGAAAGGAT GAAGGATACC CTGAGGATTG CATATCTTAC**

**TGAAGCTAAA GTCGAAAAGT TATGTGTATG GAATAATAAA ACGCCTCATG CGATTGCCGC**

**AATTAGTATG GCAAATTAA**G ATATAAAAAA GCCCACCTCA GTGGGCTTTT TTGTGGTTCG

ATGATGAGAA GCAACCGTTT TGCCCAAACA TGTATTACTG CAAGTATGAT GTTTTTATTC

CACATCCTTA GTGCGTATTA TGTGGCGCGG CATTATGTTG AGGGGCAGTC GTCAGTACCA

TTGCGCCAGC ACTGACGGCC TCACTTGCAG CAGAACGTGG GCAGCTTGCT GAATCGTTCT

GCAAGAGTGA GCCCGTAACA TAATGGCGTA TAATACGCAT TAAGGCGGTA TGTCATTTCG

GTATGTCAAA AATGACATAA TTCGATTTAT TCTGATTCCA GCCGTCCGCC GCAGTCATCA

GCTTCGCTGA TGCGAGGAGA CGGAATTTCT ACAGGTTCTA TTGAGACAGC GGCCGCTGTG

AGCTTAATTG TCTCACCTCT ATACTGCGAC AGCGGCAGGT GAGAACATAA GCGACGTAGC

GTGCGGAGTC GCGTTGTTAG AGCCTGTCCG CTGTGGTAGA CCCCCGTCTA GTATTACGGG

GGTAAATCCC ACAGAGCCTG TGACACTCAC CTTGTATTCG CAAGCGTAGC GCGCCAGTGT

TTGAGCGCTA GCGAGTCTTG CTAAGCACCA TGATTTAAGA TGCTCTTGGT AGAATGTCTT ctxB-ig HindIII R Ch1 ctxB-ig-Uni-SacI R

ATCAGCATAC TTTCTAAAAC CATGCTTATT GCTTTTTGCT CTTCTTCATC TAACTGTTGG

ATTTTTTTTA ACCTGAGCAT AAGCTCTTGA TTTTCATCTG TGGCCCATCT TCCGCATAGT

TCATCAATTG AGATCTCCAG AGCATCTGCG ATCTTCACAA GGTTTTCCAT TGTAGGCAAA

CCTTCCCCAG ATTCGTATTT TTTGTACGAT GTTAGACTAA TTCCAATTTC ATCAGCCATT
